# Camel Milk Is a Safer Choice than Goat Milk for Feeding Children with Cow Milk Allergy

**DOI:** 10.5402/2011/391641

**Published:** 2011-06-29

**Authors:** Mohammad Ehlayel, Abdulbari Bener, Khalid Abu Hazeima, Fatima Al-Mesaifri

**Affiliations:** ^1^Section of Pediatric Allergy-Immunology, Department of Pediatrics, Hamad Medical Corporation, P.O. Box 3050, Doha, Qatar; ^2^Weill Cornell Medical College, Doha, Qatar; ^3^Department of Medical Statistics and Epidemiology, Hamad Medical Corporation, Doha, Qatar; ^4^Department of Public Health, Weill Cornell Medical College, Doha, Qatar; ^5^Deptartment Evidence for Population Health Unit, School of Epidemiology and Health Sciences, The University of Manchester, Manchester, UK; ^6^Section of Pediatric Gastroenterology, Department of Pediatrics, Hamad Medical Corporation, Doha, Qatar; ^7^Department of Pediatrics, Hamad Medical Corporation, Doha, Qatar

## Abstract

*Background.* Various sources of mammalian milk have been tried in CMA. *Objectives.* To determine whether camel milk is safer than goat milk in CMA. *Methods.* Prospective study conducted at Hamad Medical Corporation between April 2007 and April 2010, on children with CMA. Each child had medical examination, CBC, total IgE, cow milk-specific IgE and SPT. CMA children were tested against fresh camel and goat milks. *Results.* Of 38 children (median age 21.5 months), 21 (55.3%) presented with urticaria, 17 (39.5%) atopic dermatitis, 10 (26.3%) anaphylaxis. WBC was 10, 039 ± 4, 735 cells/*μ*L, eosinophil 1, 143 ± 2, 213 cells/*μ*L, IgE 694 ± 921 IU/mL, cow's milk-specific-IgE 23.5 ± 35.6 KU/L. Only 7 children (18.4%) tested positive to camel milk and 24 (63.2%) to goat milk. 6 (15.8%) were positive to camel, goat, and cow milks. Patients with negative SPT tolerated well camel and goat milks. *Conclusions.* In CMA, SPT indicates low cross-reactivity between camel milk and cow milk, and camel milk is a safer alternative than goat milk.

## 1. Introduction

In infants and young children, CMA is the most common food allergic disease [1]. Total elimination of cow milk protein from child diet and providing a suitable, nutritional, substitute supply for feeding are the only current strategies [2, 3]. Extensively hydrolyzed and soy-based formulas are the most commonly used substitutes of cow milk protein in children with CMA [4, 5]. Although their nutritional value is high, their high cost and poor palatability by some children limit the use of extensively hydrolyzed formulas. For these reasons, there has been continuous search for other nonbovine, mammalian milks as a replacement of cow milk. These trials included milk of sheep, goat, ass or donkey, mare, and buffalo [6–9]. Unfortunately, it has been demonstrated, by several studies, that children with CMA develop allergy to the milk proteins of these mammalian milks due to some similarity between the proteins of these mammalian milks and that of the cow milk [10].

Goat milk has been tried but it seems it is not an appropriate alternative supply for children with IgE-mediated CMA as has been demonstrated by skin testing, CAP test, and challenge tests [6]. 

In this study, part of a prospective cohort study on camel milk, we wished to (a) find out the cross-reactivity between camel milk, goat milk, and cow milk in children with CMA, and (b) determine whether camel milk could be regarded as a safer alternative to goat milk in feeding these children. 

## 2. Subjects and Methods

### 2.1. Subjects

This prospective cohort study was conducted between April 2007 and April 2010 on 38 children less than 14 years, who were referred to the Pediatric Allergy-Immunology Clinics at Hamad Medical Corporation with symptoms related to CMA. The inclusion criteria required (i) history highly suggestive of CMA, (ii) elevated cow milk protein-specific IgE ≥0.35 IU [3], and (iii) positive skin prick test to cow milk protein. However the gold standard for diagnosing CMA is a “double blind placebo controlled food challenge” (DBPCFC) [11, 12]. Exclusion criteria included severe skin disease that precludes skin testing; persistent daily need for oral antihistamines, immunosuppressive drugs; severe cardiovascular, renal, debilitating disease (e.g., terminal malignancy), marasmic kwashiorkor, or respiratory disease, or current use ß-blockers. Data was collected using a structured interview and a standardized questionnaire. The questionnaire also included family history of allergies in parents, siblings, and grandparents.

The study was approved by the Hamad General Hospital, Hamad Medical Corporation. All human studies have been approved by the Research Ethics Committee and have been performed in accordance with the ethical standards laid down in the 1964 Declaration of Helsinki. All children were included in this study after informed consent from parents. We evaluated camel milk allergy by using skin prick test with camel milk. 

### 2.2. Skin Prick Tests (SPT) and Reagents

Milks of goat and camel were collected from local farms. They were sterilized at 100 to 120°C for ten to 20 minute [13, 14]. After cooling down, they were stored and placed at 4°C and used within 3 days. Skin prick tests were performed following the *Updated Practice Parameter of Allergy Diagnostic Testing *[15]. Tests were performed on the volar surface of the forearm with undiluted goat and camel milk allergen using a sterile lancet (Stallergen, Paris, France). A 50% glycerin/saline solution and HCL histamine solution at 10 mg/mL were used as negative and positive controls, respectively. SPT were read after 15–20 minutes, and test was considered positive if the mean diameter of the tested allergen should be at least 3 mm and greater than the histamine control. All patients had SPT with homogenized cow milk. 

### 2.3. Laboratory Workup

All patients had blood tests for CBC, total serum IgE, and cow milk-specific IgE measured by RIDA AllergyScreen Panel 3 test kit (R-Biopharm AG, Darmstadt, Germany).

## 3. Statistical Analysis

The data was analyzed using the Statistical Packages for Social Sciences (SPSS), Window version number 17. With frequency distributions, one- and two-way tabulations were obtained. Chi-square analysis was performed to test for differences in proportions of categorical variables between two or more groups. In 2 × 2 tables, the Fisher's exact test (two-tailed) replaced the chi-square test if the assumptions underlying chi-square violated, namely, in case of small sample size and where the expected frequency is less than 5 in any of the cells. The level *P* < .05 was considered as the cut-off value for significance.

## 4. Results

Table 1 shows the demographic characteristics of 38 children with confirmed cow milk allergy who were skin tested against camel milk and goat milk. Their median age was 21.5 months, range 4–126 months. Males outnumber female by a ratio of 1.92 : 1. 61% (23 of 38 children) of them had been breastfed for more than 12 months. Family history of allergy was positive in 26 children (68.4%). 

The socioeconomic features of these patients are shown in Table 2. Most of them are members of educated, urban-living families of high income. 

Table 3 shows the clinical presentation of their allergic disease and laboratory workup. The most common (29 children, 76.3%) allergic presentations were acute urticaria and anaphylactic reactions. Peripheral blood eosinophilia (absolute blood count ≥500 cells/*μ*L) was noticed in 19 patients (50%) and an elevated total serum IgE in 33 patients (86.8%). 

**Figure 1:**
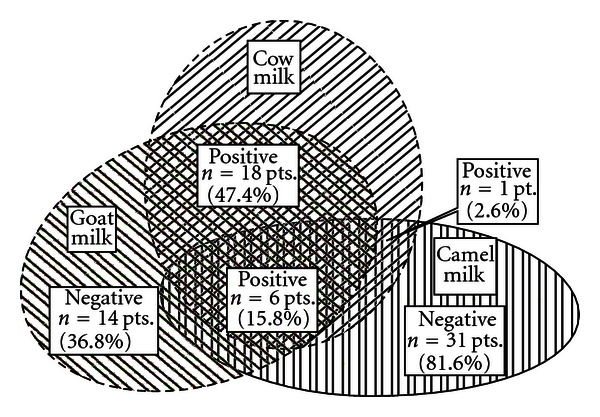
Venn diagram of SPT and cross-reactivity status among camel milk, goat milk, and cow milk in 38 children studied.

Figure 1 illustrates SPT cross-reactivity between camel milk and cow milk, goat milk and cow milk, and also between camel milk and goat milk. Only 7 patients (18.4%) had positive SPT to camel milk, while 24 (63.2%) children tested positive to goat milk. There were 6 children (15.8%) who tested positive to camel, cow and goat milks. All children (31 children) with negative SPT to camel milk tolerated (two feeds of at least 50 mL few day apart) camel milk without any reaction. At least there are 7 children (>12 months old) who have been daily taking camel milk as their main milk for more than 12 months. Unfortunately, parents of children with positive camel milk/and goat milk tests refused an offered oral challenge tests.

## 5. Discussion

In this clinical pilot study in children with CMA, since these results are based on SPT, cross-sensitization between camel milk and cow milk (7 children, 20%) is lower than that between goat milk and cow milk (24 children, 63.2%). In addition, there is cross-sensitization between camel milk and goat milk (6 children, 15.8%). This is most likely to be due to lower molecular similarity between camel milk and cow milk than that between goat milk and cow milk [10]. In addition, milk of camels and dromedaries do not contain *Bos d 5*, a whey protein abundant in cow milk [3]. Therefore children allergic to beta-lactoglobulin are safer to consume camel's milk [3].

To the best of our knowledge, this clinical study is the first study to determine the SPT-based cross-reactivity between camel milk, goat milk, and cow milk in children with CMA. Previously, all trials were done on goat milk and cow milk, and even those trials that involved camel milk did not include goat milk [16]. 

Bellioni-Businco study on 26 children with CMA revealed that all children had positive skin test, specific IgE titers to goat milk, and most of them (24 of 26 children) had positive challenge tests to goat milk, making goat milk an inappropriate substitute for children with CMA [6]. Infante Pina et al. [17] demonstrated through radioallergosorbent assay (RAST), specific IgE, skin prick and challenge tests that only 25% of the patients showed adequate immediate and late oral tolerance and had negative results of immunological tests for adverse reactions, indicating a cross-reactivity between both proteins *in vivo* and *in vitro*. They recommended that goat milk should not be given to patients with cow milk allergy without investigation of possible tolerance by a specialist [17]. Its use has been reported that it could lead to anaphylactic reaction in children with CMA [18]. Goat milk seems to cross-react not only with cow milk proteins, but also with that of other mammalian milks such as sheep [19–21]. In this aspect, goat milk seems to be as allergic as cow milk [5]. The current study confirms that camel milk is an alternative to goat milk and cow milk. 

In contrast, *in vitro* studies revealed low or absent immunological cross-reactivity between camel milk proteins and cow milk proteins. Youcef et al. [22] *in vitro* demonstrated that there is cross-reaction between some camel (dromedary) milk proteins with cow milk proteins. El-Agamy et al. [23], using specific antisera and applying immunoblotting analysis on 40 children with CMA showed absence of immunological cross-reactivity between camel and cow milk proteins, and absence of camel milk-specific IgE antibodies in the serum of these children. Furthermore, camel milk has been used in treatment of some food allergies in a small sample size of children [24]. Structurally, the degree of milk protein similarity between camel milk and that of cow milk is low (60%) compared with that between goat milk and cow milk (87.6%) [10].

Unfortunately, there has been no, *in vivo* or *vitro,* studies published on immunological cross-reactivity/sensitization between goat milk and camel milk in particular. In Katz study [15], it would be difficult to tell if there is cross-creativity to goat milk in the 2 patients (25%) with positive skin test to camel milk since the study did not include goat milk. To the best of our knowledge, our study presents, for the first time, that there is cross-sensitization between camel milk and goat milk in children with CMA as shown by skin prick test.

The effectiveness of our study is that it is a cohort, prospective, clinical study. We applied stringent inclusion criteria and recognized diagnostic parameters against cow and camel milk testing. It also clinically confirmed negative SPT reactivity to camel milk and goat milk by taking these milks. 

However our study has some limitations. Unfortunately, parents of children with positive camel milk tests refused an offered oral challenge tests knowing possibility of high risk of allergic reaction from goat milk and lack of evidence on the risk of reaction to camel milk. Therefore, subjects with positive skin prick for camel milk were not evaluated by a formal, supervised, titrated, oral, challenge test (e.g., DBPCCFCs). It also did not include any *in vitro* part to define the immunological similar/dissimilar parts of the camel milk, cow milk, and goat milk protein using the sera of our subjects.

## 6. Conclusions

In summary, this study suggests that camel milk can be regarded as a safer substitute than goat milk in most of children with symptoms suggestive of CMA. It is essential to confirm camel milk tolerability by titrated, supervised, oral challenge test in those with SPT and monitor the growth parameters of these children on camel milk.

## Figures and Tables

**Table 1:** Basic demographic characteristics of patients studied (*N* = 38).

Variable	CMA children
Patients (%)	38 (100%)
Age (months, median)	21.5
Sex:	
Males	25 (65.8%)
Females	13 (34.2%)
BMI (mean ± SD)	16.7 ± 2.5
Birth orders:	
First	11(28.9%)
2nd-3rd	17 (44.7%)
Duration of breast feeding:	
0–6 mo.	3 (7.9%)
7–12 mo.	12 (31.6%)
13–18 mo.	14 (36.8%)
19–24 mo.	9 (23.7%)
Family history of allergy:	
Allergic rhinitis	10 (26.3%)
Asthma	13 (34.2%)
Atopic dermatitis	6 (15.8%)

**Table 2:** Socioeconomic characteristics of 38 cow milk allergic children.

Variable	Patients *n* (%)
Father's level of education:	
≤ Secondary	9 (23.7)
University	29 (76.3)
Mother's level of education:	
≤ Secondary	11 (28.9)
University	27 (71.1)
Father's occupation:	
Professional	13 (34.2)
Businessman/Entrepreneur	16 (42.1)
Mother's occupation:	
Professional	2 (5.3)
Businesswoman/Entrepreneur	5 (13.2)
Housewife	31(81.6)
Place of residence:	
Urban	28 (73.7%)
Semi-urban	10 (26.3%)
Type of house:	
Flat	15 (39.5)
Villa	23 (60.5)
Family monthly income (Qatari Riyals*):	
<5,000	3 (7.9)
5000–9,999	10 (26.3)
10,000–14,999	12 (31.6)
≥15,000	13 (34.2)

*One US Dollar = 3.65 Qatari Riyals.

**Table 3:** Clinical presentation and laboratory results of cow milk allergic children (*n* = 38).

Variable*	
Clinical Presentation	*n* (%)
Anaphylaxis	10 (26.3)
Atopic dermatitis	15 (39.5)
Chronic diarrhea	1 (2.6)
Chronic vomiting	6 (15.8)
Poor weight gain	8 (21.1)
Urticaria	21 (55.3)
Others	4 (10.5)
Laboratory Workup	mean (±SD)
WBC (cells/*μ*L)	10,039 ± 4,735 (Range 7990–28,000)
Absolute eosinophil count (cells/*μ*L)	1,142 ± 2,213
Total IgE (IU/mL)	694 ± 921
	(Range 4–2,865)
Cow milk-specific IgE (KU/L)	23.48 ± 35.6
	(Range1–100)

## Abbreviations

CMA: Cow milk allergy
SPT: Skin prick test.
